# Combinatorial drug screening identifies compensatory pathway interactions and adaptive resistance mechanisms

**DOI:** 10.18632/oncotarget.938

**Published:** 2013-04-10

**Authors:** Mark Axelrod, Vicki L. Gordon, Mark Conaway, Adel Tarcsafalvi, Daniel J. Neitzke, Daniel Gioeli, Michael J. Weber

**Affiliations:** ^1^ Department of Microbiology, Immunology and Cancer Biology, University of Virginia, Charlottesville, USA; ^2^ Department of Public Health Sciences, University of Virginia, Charlottesville, USA; ^3^ Department of Internal Medicine, University of Arkansas for Medical Sciences, Little Rock, USA; ^4^ Medical Scientist Training Program, Medical University of South Carolina, Charleston, USA

**Keywords:** Targeted therapies, Compensatory signaling, Pathway interactions, Crosstalk, Feedback inhibition

## Abstract

Constitutively activated signaling molecules are often the primary drivers of malignancy, and are favored targets for therapeutic intervention. However, the effectiveness of targeted inhibition of cell signaling can be blunted by compensatory signaling which generates adaptive resistance mechanisms and reduces therapeutic responses. Therefore, it is important to identify and target these compensatory pathways with combinations of targeted agents to achieve durable clinical benefit. In this report, we demonstrate the use of high-throughput combinatorial drug screening as a discovery tool to identify compensatory pathways that generate resistance to the cytotoxic effects of targeted therapy. We screened 420 drug combinations in 14 different cell lines representing three cancer lineages, and assessed the ability of each combination to cause synergistic cytotoxicity. Drug substitution studies were used to validate the functionally important drug targets. Of the 84 combinations that caused robust synergy in multiple cell lines, none were synergistic in more than half of the lines tested, and we observed no pattern of lineage specificity in the observed synergies. This reflects the plasticity of cell signaling networks, even among cell lines of the same tissue of origin. Mechanistic analysis of one novel synergistic combination identified in the screen, the multi-kinase inhibitor Ro31-8220 and lapatinib, demonstrated compensatory crosstalk between the p70S6 kinase and EGF receptor pathways. In addition, we identified BAD as a node of convergence between these two pathways that may be playing a role in the enhanced apoptosis observed upon combination treatment.

## INTRODUCTION

Dysregulation of cell signaling networks caused by mutationally altered proteins drives the biological transformations that constitute the hallmarks of cancer [1]. Identification of these oncogenic drivers provides an opportunity for therapeutic intervention using targeted agents [2]. Unfortunately, in most advanced cancers the therapeutic responses to targeted agents are partial and not durable [3]. Increasing evidence points to the robustness of cell signaling networks as a substantial component of both primary and acquired resistance to targeted therapies [4,5].

Resistance can be caused by the activity of alternative signaling pathways that compensate for the pathways being inhibited by the therapeutic agent (reviewed in [6,7]). Due to the robustness of the signaling networks, it will be necessary to inhibit not only the primary drivers of oncogenesis, but also one or more of the compensatory signaling modules. We term these secondary drivers “backseat drivers”. Identifying these secondary targets represents a major challenge in developing rational combinations of targeted drugs. We previously reported that some compensatory responses could be identified by analysis of phosphoproteomic or gene expression changes that occur in response to single drug treatment [4]. However, the diversity of these changes and our limited knowledge of how signaling pathways are laterally linked into functional networks makes it difficult to predict what the most appropriate target(s) would be for co-inhibition. Moreover, the limited repertoire of targets utilized for drug development [8] makes it difficult to rationally construct drug combinations that could have near-term clinical utility.

To identify targetable compensatory pathways that could guide the construction of potentially useful drug combinations, we have performed robotic screens with a library of targeted agents, singly and in combination, and identified combinations that caused synergistic cytotoxicity. We focused on synergistic combinations because they point to mechanistic linkages between the signaling pathways, and also because of the possibility of improved therapeutic index *in vivo*. In assembling the library we emphasized use of FDA-approved drugs, drugs in clinical trials, and tool compounds that inhibit equivalent and validated targets. We [9] and others [10] have utilized panels of B-Raf, N-Ras and double wild-type melanomas and shown that unexpected synergistic combinations could be identified by screening combinations of targeted agents. In this communication, we expand and refine the screening approach using a panel of epithelial cancer cell lines. Drug combinations identified using this screening technique were then tested in a series of follow-up experiments to verify the targets of the inhibitors, and determine a mechanism of compensatory interaction between the pathways being inhibited. We identified expected combinations (e.g. PI3 kinase and EGF receptor pathway inhibitors), which provided a validation of the approach. We also identified novel, unexpected combinations, some of which may have potential for clinical development. Mechanistic analyses of one of these novel combinations, a p70S6 kinase inhibitor and an EGF receptor inhibitor, identified BAD phosphorylation as a terminal node of convergence of the pathways being inhibited

## RESULTS

Chemical genetic synthetic lethal screening identifies drug combinations causing synergistic cytotoxicity. To identify pathways that can compensate for blockade of an oncogenic target, we performed a combinatorial synthetic lethal screen utilizing a library of sixty targeted small molecule inhibitors. Some of the inhibitors are approved drugs, some are in clinical development and others are tool compounds. The library contained protein kinase inhibitors, inhibitors of other enzymatic processes such as deacetylation, methylation and dephosphorylation, and also general chemotherapeutic agents. Each of these inhibitors was tested in combination with seven “primary” inhibitors, which were selected based on a literature search of commonly drugged targets in various cancers. This approach gave us a matrix of 420 drug combinations, which were tested in fourteen cancer cell lines, representing three distinct epithelial cancer lineages (prostate cancer (CaP), bladder cancer (BC), and head and neck cancer (HNC)). Three CaP cell lines (LNCaP, C4-2, and RV-1) tested are known to express and be dependent on the androgen receptor [11-13]. Since androgen deprivation is a standard of care for prostate cancer patients, we assessed the responses of these cell lines in both the presence and absence of R1881, a synthetic androgen, in order to better recapitulate the *in vivo* conditions associated with therapy of this type of cancer.

**Figure 1:**
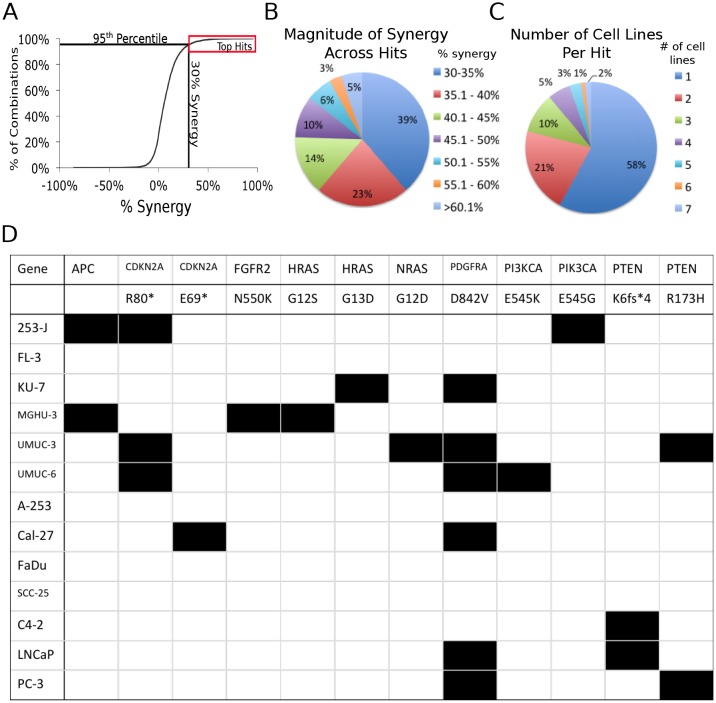
Screening with small molecule inhibitors identifies combinations of drugs that cause synergistically enhanced cytotoxicity A. 420 drug combinations were tested in 13 cells lines (plus 3 additional conditions in which the prostate cancer cell lines expressing the AR were treated with R1881). The cell lines are organized as follows: Bladder Cancer Cell Lines : A. 253-J B. FL-3 C. KU-7 D. MGHU-3 E. UMUC-3 F. UMUC-6. Head and Neck Cancer Cell Lines G. A253 H. Cal27 I. FaDu J. SCC-25. Prostate Cancer Cell Lines K. C4-2 L. C4-2 + R1881 M. LNCaP N. LNCaP + R1881 O. PC-3 P. RV-1 Q. RV-1 + R1881. The red box indicates all the inhibitor combinations that are above the 30% synergy cut-off point. B. The distribution of the magnitude of the synergistic effect across all combinations above the 95^th^ percentile cutoff. C. The number of cell lines each combination caused synergistic cytotoxicity in above the 95^th^ percentile cutoff. D. Oncomap analysis of common oncogenic mutations in the cell lines tested in the screen. Only mutations that occurred in at least one cell line are listed. A black box indicates that mutation was present in the indicated cell line.

The levels of cytotoxicity generated by each individual drug treatment and drug combination were assayed using alamarBlue, and synergy was assessed by the Bliss independence method [12]. The differences between the Bliss model predictions for additivity and the actual level of cytotoxicity, a value we term the ‘percent synergy,’ were determined for every drug combination tested in each cell line. These data were plotted (Figure 1A) and a cutoff was set for the top 5%, which corresponded to 30% synergy. (i.e. the actual amount of cytotoxicity was at least 30% greater than an additive interaction predicted by Bliss Independence). The majority of the “hits” (39%) caused between 30 – 35% synergy. As the magnitude of the synergy increased, the proportion of hits decreased (Figure 1B). Strikingly, not one of these combinations resulted in synergistic cytotoxicity in more than half the cell lines, with the majority of hits (58%) occurring in only one cell line (Figure 1C). In addition, no combination that resulted in synergy in more than 2 cell lines was lineage specific. The diversity of these responses to treatment undoubtedly reflects the diverse architecture of the underlying cell signaling networks, and the ways the genetic context of these cells alters responses to individual targets. Analysis of common oncogenic mutations (Figure 1D) did not reveal any underlying genetic changes that could be used to predict sensitivity to any of the synergistic drug combinations.

**Figure 2:**
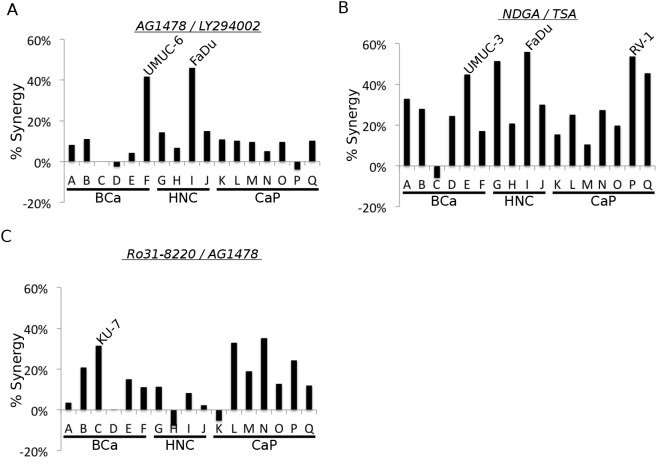
Synergistic drug combinations selected for follow-up investigation. A. The calculated ‘% synergy’ levels for the combination of EGF receptor and PI3 kinase inhibitors used in the screen. B. The calculated ‘% synergy’ levels for the combination of NDGA and TSA from the screen. C. The calculated ‘% synergy’ levels for the combination of Ro318220 and AG1478 from the screen.

We then chose three combinations for further study. The combination of AG1478/LY294002 was selected because the synergistic interaction between EGF receptor and PI3 kinase inhibitors has been well documented in other model systems (ex. [14]), making this combination a useful proof-of-principle for the screening and validation methodology (Figure 2A)). The combinations of NDGA/TSA (5-Lipoxygenase inhibitor/HDAC inhibitor) and AG1478/Ro318220 (EGFR inhibitor/multi-kinase inhibitor) were selected due to their occurrence in multiple cell lines and the novelty of the interactions between the pathways that these drugs inhibit (Figures 2B and 2C).

**Figure 3:**
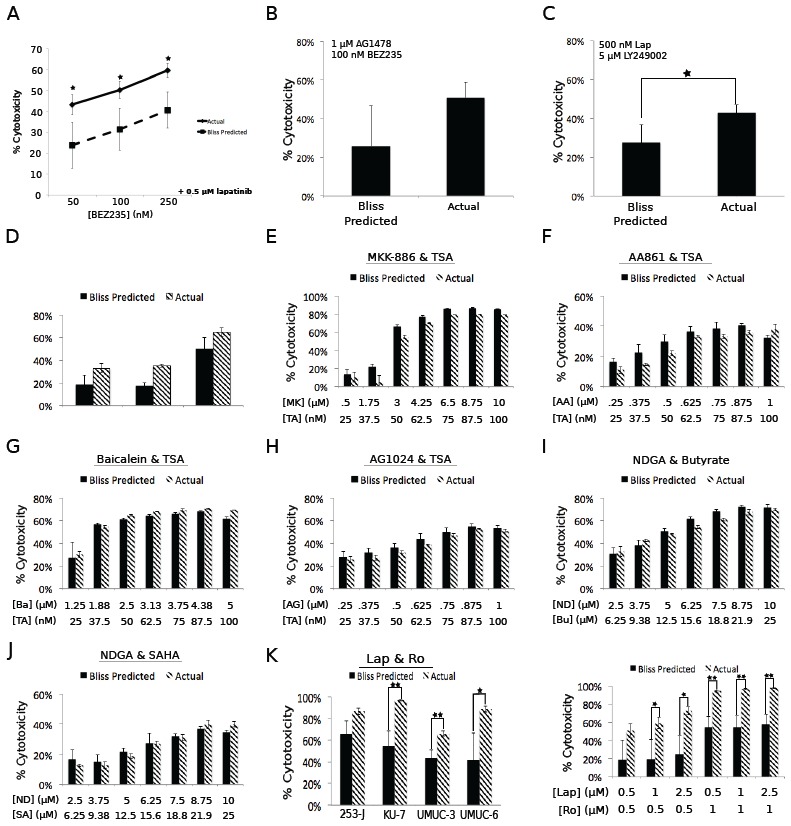
Drug substitutions can validate the targets that must be inhibited to achieve synergistic cytotoxicity. A-C. UMUC-6 cells were treated for 72 hours with the indicated drugs and concentrations. BEZ235 (A&B) was used as a substitute for LY294002. Lapatinib (A&C) was used as a substitute for AG1478. D. UMUC-3, FaDu, and RV-1 were treated for 72 hours with NDGA and TSA and synergy was assessed. E-I. UMUC-3 cells were treated for 72 hours with the indicated drugs and concentrations. MKK-886 (FLAP inhibitor) (E), AA-861 (5-LOX inhibitor) (F), Baicalein (12- and 15-LOX inhibitor) (G), and AG1024 (IGFR inhibitor) (H) were used to substitute for NDGA in combination with TSA (TA). Sodium Butyrate (I) and SAHA (J) were used to substitute for TSA (at concentrations that caused equivalent histone acetylation) in combination with NDGA (ND). K. Multiple bladder cancer cell lines were treated with lapatinib (substitute for AG1478) and Ro31-8220 for 72 hours. L. KU-7 cells were treated for 72 hours with the indicated concentrations of Ro31-8220 and lapatinib (as a substitute for AG1478). Bars represent the mean and error bars represent the standard deviation of three technical triplicates. Relative cell number was assessed using alamarBlue. The Bliss Predicted values were generated as described in the Methods section.

Drug substitution experiments confirm the targets necessary for synergistic cytotoxicity. Small molecule drugs often inhibit the activity of proteins other than their primary target [15]. In order to test whether these “off-target” effects were responsible for the synergy observed in the screen, we performed follow-up growth assays using other inhibitors of the same putative targets as the drugs used in the initial screen. For the combination of AG1478/LY294002, we used lapatinib or gefitinib as substitute EGF receptor inhibitors and NVP-BEZ235 or wortmannin as substitute PI3 kinase inhibitors. We tested these compounds in UMUC-6 cells. A range of lapatinib concentrations combined with 100 nM BEZ235 resulted in significant synergy, confirming the screen results (Figure 3A). In addition, the combinations of AG1478/BEZ235 (Figure 3B) and lapatinib/LY294002 (Figure 3C) were able to recapitulate the synergy observed in the screen. The combinations of gefitinib (EGF receptor)/LY294002 and wortmannin (PI3 kinase)/AG1478 also produced enhanced cytotoxicity (Supplemental Figure S1). These results confirm the identification of EGF receptor and PI3 kinase as the functional targets responsible for the synergistic small molecule interactions.

We next examined the combination of NDGA/TSA. In order to confirm the synergy observed in the screen, we tested this combination in three cell lines; UMUC-3, FaDu, and RV-1. This combination reproducibly resulted in significant synergy in UMUC-3 and FaDu and trended towards synergy in RV-1 (Figure 3D). However, in contrast to the studies with the EGFR/PI3K inhibitors, drug substitutions failed to validate the putative target for either agent. The 5-Lipoxygenase Activating Protein (FLAP) inhibitor MKK-886, the 5-Lox inhibitor AA-861, the 12- and 15-Lox inhibitor baicalein, and the IGFR inhibitor AG1024 failed to replicate the synergizing properties of NDGA (Figure 3E-3H for UMUC-3 and Supplemental Figures S2 A-D and S3 A-D for FaDu and RV-1, respectively). We also tested whether other HDAC inhibitors could substitute for TSA to cause synergy in combination with NDGA including SAHA (Vorinostat) and sodium butyrate. These inhibitors were tested at both concentrations that caused increases in histone H3 acetylation similar to the concentrations of TSA that resulted in synergistic cytotoxicity (data not shown), as well as concentrations that were comparable or exceeded those found in the plasma of human patients treated [16,17] with these drugs (Figure 3 I and J, Supplemental Figures S2I, S2J, S3I and S3J). Neither of these inhibitors was able to cause synergy in combination with NDGA (Figure 3I-3J for UMUC-3 and Supplemental Figures S2 E-F and S3 E-F for FaDu and RV-1, respectively). Thus, the functionally important targets for the NDGA/TSA synergy remain unknown.

Lastly, to confirm the necessity of EGF receptor inhibition for synergy in combination with Ro31-8220, we again substituted lapatinib for AG1478. We found that lapatinib caused significant synergy in three bladder cancer lines (KU-7, UMUC-3 and UMUC-6) and trended towards significance in a fourth (253-J) (Fig. 3K). We also tested nine concentration combinations of lapatinib/Ro31-8220 in KU-7 cells and found significant synergy over a range of concentrations of both drugs (Figure 3L).

**Figure 4:**
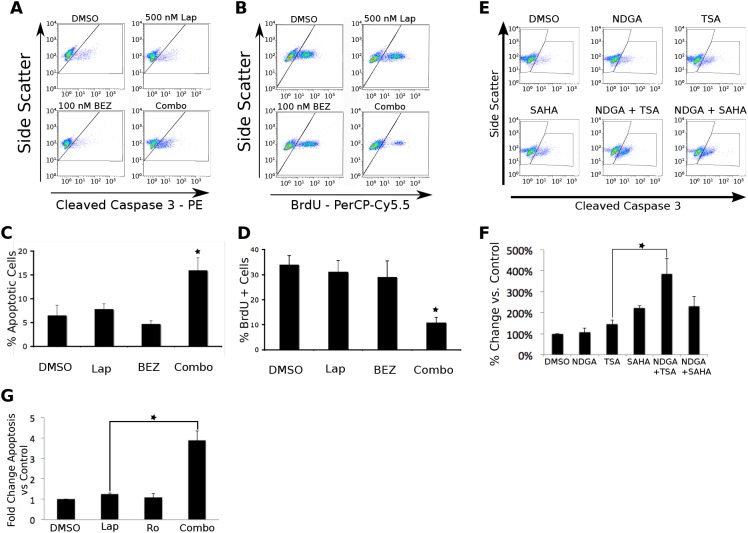
Flow cytometry analysis reveals the biological outcomes of synergistic drug combinations identified by synthetic lethal screening. A and B. UMUC-6 cells were treated for 72 hours with either DMSO control 500 nM lapatinib, 100 nM BEZ235 or the drug combination. C and D. Quantification of the flow cytometry data from three independent experiments. E. UMUC-3 cells were treated with vehicle, 2.5 μM NDGA, 75 nM TSA, 1 μM SAHA, the combination of NDGA and TSA, or the combination of NDGA and SAHA for 72 hours. F. Three independent experiments were quantified. G. KU-7 cells were treated with DMSO, 500 nM Ro, 500 nM lapatinib, or the combination for 24 hours. Three independent experiments were quantified. Apoptosis was assessed by caspase 3 cleavage or PARP cleavage. Proliferation was assessed by BrdU incorporation. Both were assayed by flow cytometry. The bars represent the mean of three independent experiments and the error bars represent the SEM. The * indicates a p-value < .05. The ** indicates a p-value < .001.

Flow cytometry analysis determines the biological effects of synergistic drug combinations. To identify the biological endpoints of the synergistic cytotoxicities, we measured effects on apoptosis, proliferation, and cell cycle progression by flow cytometry. In UMUC-6 cells, treatment with 500 nM lapatinib or 100 nM BEZ235 alone had minimal effects on apoptosis and proliferation, but treatment with the combination caused a synergistic increase in apoptosis and decrease in proliferation (Figure 4 A-D). Similarly, KU-7 cells treated with lapatinib or Ro31-8220 and UMUC-3 cells treated with NDGA or TSA demonstrated little change in the levels of apoptosis. In both cases, however, combination treatment resulted in a significant increase in apoptosis (Figure 4 E-G). The robust apoptosis generated by the NDGA/TSA combination makes it appealing for further study to determine whether it may be useful therapeutically. However, in agreement with data from our cytotoxicity assays, the combination of SAHA and NDGA did not result in synergistic apoptosis (Fig 4 E-F). The lack of specificity of the inhibitors makes it impossible to use them as probes of regulatory mechanisms and the inability of clinically useful HDAC inhibitors to produce synergy with NDGA makes it impossible to advance this combination towards clinical trials.

**Figure 5:**
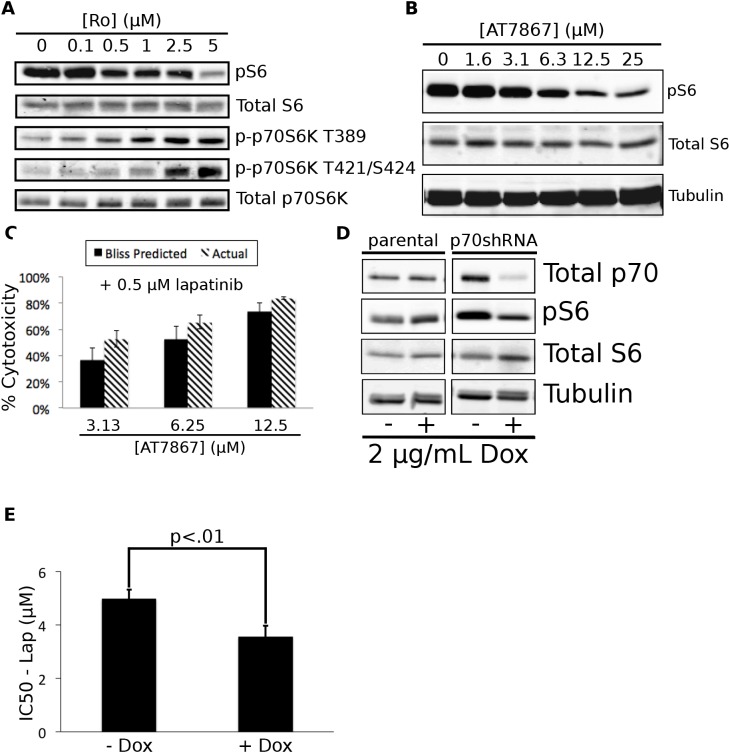
Inhibition of p70S6K mediates the synergistic effects of Ro31-8220 in combination with lapatinib. A. KU-7 cells were treated for 1 hour with the indicated concentrations of Ro31-8220. Cells were then lysed and Western blotted for the indicated phosphorylated and total proteins according to the Methods section. Representative data from three independent experiments is shown. B. KU-7 cells were treated with the indicated concentrations of AT7867 for 24 hours. The cells were then lysed and Western blotted for the indicated phosphorylated and total proteins. Representative data from three independent experiments is shown C. KU-7 cells were treated for 72 hours with the indicated concentrations of AT7867 +/- 500 nM lapatinib. Relative cell number was assessed using alamarBlue. The Bliss Predicted values were generated as described in the Methods section. D. KU-7 cells stably expressing a doxycycline-inducible p70S6K shRNA or parental KU-7 cells were treated with vehicle or 2 μg/mL doxycycline for 72 hours. The cells were then lysed and Western blotted for the indicated phosphorylated and total proteins. Representative data from three independent experiments is shown. E. KU-7 cells stably expressing a doxycycline-inducible p70S6K shRNA were treated with vehicle or 2 μg/mL doxycycline for 72 hours. The cells were then re-plated in 96 well format and treated for an additional 72 hours with the indicated concentrations of lapatinib +/- 2 μg/mL doxycycline. Lapatinib IC50 values were calculated in the presence or absence of p70S6K knockdown.

p70S6 kinase inhibition in combination with lapatinib recapitulates the effects of Ro31-8220 treatment. We selected the EGF receptor inhibitor/Ro31-8220 combination for further mechanistic analysis based on its novelty and the challenges presented in utilizing a multi-kinase inhibitor in developing combinatorial targeted therapies. Ro31-8220 is related to staurosporine and UCN-01, and was developed as a PKC inhibitor [18]. However, it inhibits the activity of a number of protein kinases including p70S6 kinase [15], which we have observed to be an important node in other synergistic drug interactions (Axelrod, et. al – under revision). To determine whether Ro31-8220 was inhibiting the activity of p70S6 kinase in KU-7 cells, we treated the cells with increasing concentrations of Ro31-8220. This treatment resulted in concentration dependent decreases in S6 phosphorylation at serines 240 and 244 (S240/S244). In contrast, activating p70S6 kinase phosphorylations at threonine 389 (T389), threonine 421, and serine 424 (T421/S424) were increased in a concentration dependent manner (Figure 5A), presumably reflecting a de-inhibition of upstream kinases by relief of feedback inhibition. These data indicate that Ro31-8220 is a direct inhibitor of p70S6 kinase enzymatic activity and does not significantly inhibit upstream pathway activity at concentrations sufficient to inhibit p70S6 kinase activation.

To determine whether p70S6 kinase inhibition was sufficient to obtain synergy with lapatinib, we tested the utility of AT7867, a structurally distinct multikinase inhibitor. AT7867 has a different target profile from Ro31-8220, however p70S6 kinase is the only published target shared by these two drugs [15,19]. As with Ro31-8220, treatment of KU-7 cells with AT7867 resulted in a concentration dependent decrease in pS6, indicating that p70S6 kinase activity was in fact being inhibited by AT7867 (Figure 5B), and the combination of AT7867 with lapatinib resulted in synergistic cytoxicity (Figure 5C). These results strengthen the link between p70S6 kinase inhibition and cell growth inhibition.

Since AT7867 inhibits kinases other than p70S6 kinase, we used RNAi to reduce p70S6 kinase expression and determined the effects of this knockdown on lapatinib sensitivity. KU-7 cells were stably infected with a lentivirus containing a doxycycline-inducible shRNA against p70S6 kinase (KU-7-p70shRNA). Treatment with doxycycline for 48 hours resulted in a 85% decrease in total p70S6 kinase levels and a 50% decrease in pS6 levels in the KU-7-p70shRNA cells, while having no effect on the parental KU-7 cells (Figure 5D). Knockdown of p70S6 kinase expression was maintained 120 hours post-doxycycline addition (data not shown). The knockdown of p70S6 kinase caused a modest but statistically significant shift in the IC50 of lapatinib, indicating that the reduction of p70S6 kinase expression sensitized these cells to lapatinib treatment (Figure 5E), and that inhibition of p70S6 Kinase is likely an essential component of the cytotoxic effects of this combination.

**Figure 6:**
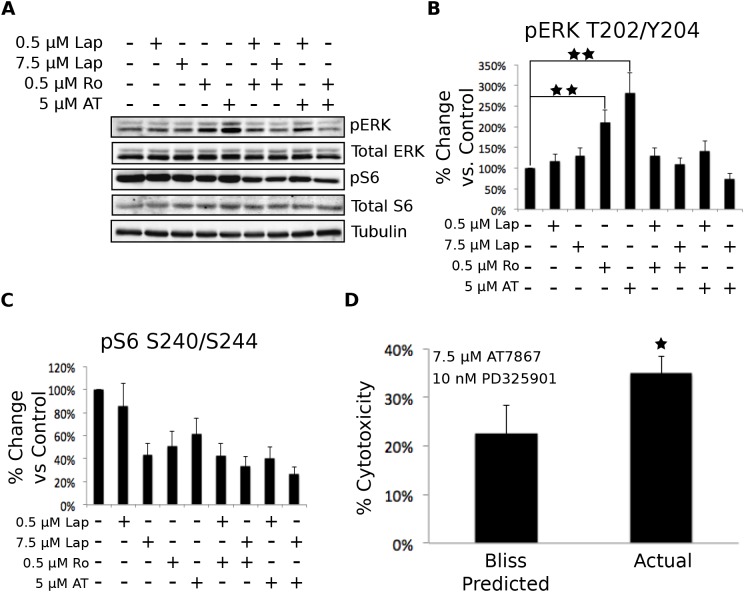
The loss of p70S6K signaling results in the compensatory activation of MEK/ERK signaling through an EGFR-dependent mechanism. A. KU-7 cells were treated with vehicle control, 500 nM or 7.5 μM Lap, 500 nM Ro, 5 μM AT, or a combination of Lap and Ro or Lap and AT for 1 hour. The cells were then lysed and blotted for Akt (pS473 and total), ERK (pT202/Y204 and total), S6 (pS240/S244 and total), and tubulin. Representative data from three independent experiments is shown. B-C. Quantification of the phosphorylated: total ratio of ERK and S6. The bars represent the mean of the three independent experiments and the error bars represent the SEM. D. Cells were treated with the indicated concentrations of AT7867 and/or PD325901 for 72 hours. Cell growth was assayed by alamarBlue. Synergistic interaction was determined using the Bliss Additivity model. The * indicates a p-value <.05. The ** indicates a p-value <.001.

p70S6 kinase inhibition leads to EGF receptor dependent compensatory signaling through ERK. To understand the molecular mechanism of the synergistic cytotoxicity from the combination of EGF receptor and p70S6 kinase inhibition, we analyzed the compensatory signals elicited by inhibition of these targets. Treatment with either Ro31-8220 or AT7867 alone resulted in an increase in ERK phosphorylation. These increases were blocked by the addition of lapatinib, indicating that the increased ERK pathway signaling was dependent upon EGF receptor activity (Figure 6 A-B). We also noted that the addition of lapatinib enhanced the inhibition of S6 phosphorylation caused by AT7867 (Figure 6 A&C). To test whether increased ERK activation was part of the compensatory pathway providing resistance to cytotoxicity in response to p70S6 kinase inhibition, we tested the effects of combined p70S6 kinase and MEK inhibition using AT7867 and PD325901, a selective MEK inhibitor. KU-7 cells were treated with these two compounds singly or in combination for 72 hours. The combination resulted in a synergistic increase in cytotoxicity (Figure 6D). These data indicate that inhibition of p70S6 kinase in KU-7 cells results in an EGF receptor-dependent, compensatory increase in ERK signaling that provided resistance to cytotoxicity caused by p70S6 kinase inhibition alone.

**Figure 7:**
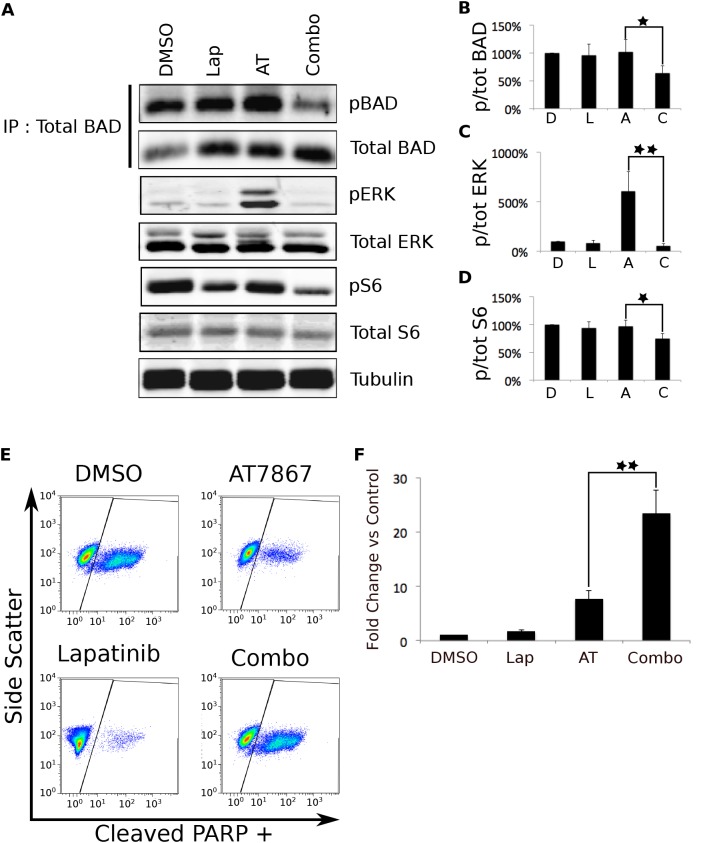
Simultaneous inhibition of EGFR and p70S6K leads to apoptosis that may be mediated through BAD hypophosphorylation. A. KU-7 cells were treated with vehicle, 7.5 μM Lap, 5 μM AT, or the combination for 1 hour. The cells were then lysed and total BAD protein was immunoprecipitated. The resulting protein was separated by SDS-PAGE and Western blotted for pS112 and total BAD. Representative data from four independent experiments is shown. The remaining whole cell lysates were Western blotted for the indicated phosphorylated and total proteins. Representative data from five independent experiments is shown. B-D. Quantification of the phosphorylated: total ratio of the indicated proteins. The bars represent the mean of the five (or, in the case of BAD, four) independent experiments and the error bars represent the SEM E. KU-7s were treated with DMSO, 5 μM AT, 7.5 μM lapatinib, or the combination for 24 hours. Apoptosis was assessed by a FACS-based PARP cleavage assay. Representative data from three individual experiments is shown. F. Quantification of the flow cytometry data from E. The * indicates a p-value <.05. The ** indicates a p-value <.001.

We further examined this compensatory signaling pathway by Western blotting of KU-7 cells treated with vehicle control, 5 μM AT7867, 7.5 μM lapatinib, or the combination for one hour. In agreement with our previous data, treatment with AT7867 caused a robust increase in ERK phosphorylation that was inhibited by lapatinib treatment (Figure 7 A&C). Lapatinib treatment also further reduced the AT7867-mediated inhibition of S6 phosphorylation (Figure 7 A&D).

Since the combination of Ro31-8220 and lapatinib resulted in synergistic apoptosis, we performed FACS analysis on cells treated with AT7867 and lapatinib to determine whether this combination produced a similar biological outcome. KU-7 cells were treated with vehicle, single drug, or the combination for 24 hours. AT7867 treatment resulted in a 7.5-fold induction of apoptosis compared to vehicle treated control cells, while lapatinib alone resulted in a modest 1.6-fold increase in cell death. The combination, however, resulted in a robust, synergistic apoptotic response that was over 23-fold higher than control cells (Figure 7 E&F).

Synergistic hypophosphorylation of BAD. Because the combination of AT7867 and lapatinib induced synergistic apoptosis, we sought to identify a pro-apoptotic signaling molecule whose regulation also was synergistically regulated. The pro-apoptotic protein BAD, which is known to be downstream of both p70S6 kinase and ERK signaling, was a logical candidate [20,21]. We immunoprecipitated total BAD protein from the same lysates that were used for analysis of ERK and S6 phosphorylation. Probing these immunoprecipitates with a BAD phospho-specific antibody, we found that while single drug treatments did not have a significant effect on BAD phosphorylation, the combination caused a synergistic decrease in phosphorylation (Figure 7 A&B), which allows it to perform a pro-apoptotic function [22].

## DISCUSSION

We and others have hypothesized that compensatory signaling pathways are induced in response to treatment with targeted cancer therapies, and that these compensatory signals can contribute to therapeutic resistance [6,7,9,10]. The compensatory pathways thus present a requirement and an opportunity for therapeutic intervention. Combined targeting of both the primary oncogenic driver as well as one or more compensatory “backseat drivers” should result in a greater anti-tumor effect than single agent treatment alone.

In this work, we present a technique for identifying combinations of primary and “backseat” drivers through the use of high-throughput combinatorial drug screening. This pathway discovery screen allowed us to find combinations of targeted therapies that produced synergistic growth inhibition. A synergistic interaction implies interconnectedness between the two pathways being inhibited. The overall concept is supported by the complementary compensatory signaling we show occurs when p70S6 kinase and EGF receptor are inhibited separately, and the synergistic cytotoxicity seen when both targets are inhibited simultaneously. The relationship between synergy and crosstalk stands in contrast to a purely additive interaction, which would result from the inhibition of two completely separate pathways.

In each of 14 cell lines we assessed the growth effects of 420 drug combinations using the Bliss model to assess synergy. Analyses of these data revealed that none of the combinations caused synergy in more than 7 cell lines, nor did any of the combinations display lineage-specific synergy. In addition, none of the common oncogenic mutations we examined in these cell lines could be used to predict synergistic responses to any of the combinations. A recent combinatorial drug study by Held, et al. identified drug combinations that were specific for sub-groups of melanoma characterized by their Raf and Ras mutational status [10]. Even within these subgroups, however, the responses to these combinations were not universal. In addition, research from our own laboratory (Roller et al., in preparation) on a panel of BRAF mutant melanoma cell lines treated with vemurafenib and lapatinib revealed varied responses in transcriptional and phosphoproteomic responses despite the common links of B-Raf mutation and synergistic cytotoxic. This diversity of responses to combination treatment strongly suggests that the unique cell signaling networks and genetic context of each cell line play a crucial role in the response of these cells to targeted therapies.

Our pathway discovery screen identified both expected and novel pathway interactions. In order to validate the screen, we first analyzed one of the expected hits: inhibition of EGF receptor combined with inhibition of PI3 kinase signaling. Using a drug substitution paradigm, we confirmed that inhibition of EGF receptor and PI3 kinase resulted in synergistic cytotoxicity irrespective of the drug used to inhibit each target. Then, using multi-variate flow cytometry, we found that dual inhibition of EGF receptor and PI3 kinase signaling pathways resulted in both a synergistic decrease in proliferation and a synergistic increase in apoptosis. These results confirmed those of the growth assays, which used the metabolic readout of alamarBlue.

We next examined two novel pathway interactions identified in the screen. The first was the combination of NDGA and TSA. NDGA, and its derivative terameprocol, are currently being evaluated in clinical trials for both leukemia and solid tumors such as prostate cancer (ex. NCT00678015, NCT00664677). The putative target for these drugs is 5-Lipoxygenase [23], although other targets have also been reported, including the IGF receptor [24]. TSA is a tool compound that has been shown to inhibit the activity of histone deacetylases. The NDGA/TSA combination produced robust synergy in seven of the 14 cell lines tested in the screen, spanning all three cancer lineages that were represented. Follow-up growth assays and flow cytometry staining for apoptosis confirmed the synergistic effect of this drug combination, making this a very attractive combination from the perspective of biological endpoints. Unfortunately, we were unable to identify the targets of these drugs whose inhibition was sufficient to result in synergy. Combining other inhibitors of the 5-Lox pathway, as well as other Lox family inhibitors and inhibitors of other published NDGA targets, with TSA did not produce synergy in any of the cell lines tested.

Similarly, the substitution of HDAC inhibitors that are either clinically approved (SAHA/Vorinostat, Valproic acid, Sodium butyrate) or in clinical trials (Panobinostat) also failed to recapitulate the synergy observed with the NDGA/TSA combination. The inability of SAHA to substitute for TSA was particularly surprising, given the structural similarities between the two compounds [25]. The failure of these drugs to substitute for TSA indicates that, even though each is a “HDAC inhibitor” it is likely that each has a different profile of functionally significant molecular targets. Over fifty non-histone protein substrates of HDAC family members have been identified [26], making the functionally significant target(s) for synergy and cytotoxicity difficult to identify. Since we were unable to find a drug that is FDA-approved or being used in clinical trials to substitute for TSA, nor were we able to identify the functional target of NDGA, we were unable to move forward with the study of this combination, despite its robust preclinical effectiveness. These findings highlight the value of the drug substitution paradigm in validation of hits obtained by small molecule screens.

The third combination identified in the screen that we chose to study further was an EGF receptor inhibitor combined with Ro31-8220, a multi-kinase inhibitor. Based on the published target profile for this compound, we tested whether inhibition of p70S6 kinase was sufficient to produce synergy in combination with EGF receptor inhibition. Using drug substitutions and RNAi, we demonstrated that inhibition of p70S6 kinase can sensitize cells to treatment with an EGF receptor inhibitor, although the synergy produced by this combination was not as robust as when Ro31-8220 was used. This is likely due to the effects of inhibition of other targets such as p90RSK by Ro31-8220 that contribute to the cytotoxic effect of the drug. Despite the differences in synergy as assessed by growth assays, both combinations produce robust apoptosis as measured by flow cytometry.

Understanding the mechanism of crosstalk between two pathways whose inhibition leads to synergy is important because it can lead to the identification of nodes between the pathways, which may prove to be useful drug targets themselves. We noted that treatment with either Ro31-8220 or the p70S6 kinase inhibitor AT7867 resulted in an increase in the levels of phosphorylated ERK. Addition of lapatinib to treatment with either of these drugs, however, completely blocked the compensatory ERK increase. Using the selective MEK inhibitor PD325901, we demonstrated that blocking this increase in ERK signaling is sufficient to cause synergy in combination with p70S6 kinase inhibition. Therefore, this EGF receptor-dependent ERK activation represents a compensatory pathway providing resistance to apoptosis upon p70S6 kinase inhibition.

The mechanism by which p70S6 kinase inhibition results in ERK activation is unknown, but it is possible that the effect is due to the relief of a p70S6 kinase-mediated negative feedback loop. p70S6 kinase is known to negatively regulate IRS-1 through a feedback phosphorylation and inhibition of p70S6 kinase results in increased IGF receptor/PI3 kinase/AKT signaling [27]. It is possible that a similar mechanism is at play in this context, although further studies will need to be performed to determine whether this feedback mechanism is occurring in this context.

While upstream feedback loops may represent one level of crosstalk between p70S6 kinase and EGF receptor/ERK signaling, another level exists at the point of pathway convergence. Both ERK, through the activity of its downstream effector p90RSK, and p70S6 kinase can phosphorylate and thereby deactivate the pro-apoptotic protein BAD [20,21]. These phosphorylations lead to the binding of and sequestration by 14-3-3 chaperone proteins. This prevents BAD from translocating to the mitochondrial membrane where it can effect its pro-apoptotic functions [28]. While treatment with either an inhibitor of p70S6 kinase or EGF receptor had little effect on BAD phosphorylation, combination treatment resulted in a significant decrease in BAD phosphorylation. These data are in agreement with our findings that the combination treatment results in increased apoptosis. She, et al. also identified Bad as a node of convergence between EGFR and AKT signaling in cells lacking functional PTEN [21]. In contrast, three out of the four cell lines in our study that displayed sensitivity to the combination of EGFR and p70S6K co-inhibition contained functional PTEN. Together, these results suggest that Bad may be a potential node regulating cell survival in multiple genetic contexts, further increasing its potential utility as a therapeutic target.

In this study, we have shown that a combinatorial drug screening technique can identify pathways that provide compensatory signaling for each other, leading to resistance to single drug treatments. By studying the effects of single drug treatment on the compensatory pathway, we have identified a mechanism through which the pro-apoptotic protein BAD remains phosphorylated and inactivated, despite p706 kinase inhibition. This is due to the activation of EGF receptor/ERK signaling, possibly as a result of the relief of a negative feedback loop upon loss of p70S6 kinase activity. Dual inhibition of these two pathways results in decreased BAD phosphorylation and synergistically increased apoptosis. In the context of patient care, these results point to the need for the analysis of individual tumors post-treatment, especially in cases where resistance to a single targeted agent has arisen. These analyses will likely provide information that can be used to identify the compensatory pathways that are leading to resistance and can allow for rationally designed combination therapies that will improve patient outcome. Additionally, the identification of downstream nodes, such as BAD, may lead to new drug targets that could be as effective, or even more effective than targeting upstream network components.

## MATERIALS AND METHODS

### Cell lines and reagents

UMUC-6, UMUC-3, KU-7, 253-J, FL-3, and MGHU-3 bladder cancer (BC) cells were from Dr. Dan Theodorescu; FaDu, SCC-25, Cal27, and A253 head and neck cancer (HNC) cells from Dr. Chris Thomas; LNCaP, C4-2, and PC-3 prostate cancer (CaP) cells were from Dr. Leland Chung and CWR22-RV-1 (RV-1) were from Dr. Thomas Pretlow. Cells were maintained in MEM supplemented with 1 mM sodium pyruvate, 0.1 mM MEM non-essential amino acids and 5% FBS (253-J, UMUC-6 and KU-7), MEM supplemented with 1 mM sodium pyruvate and 5% FBS (UMUC-3), MEM supplemented with 10% FBS (MGHU-3), Ham's/F12 1:1 supplemented with 5% FBS (FL-3), DMEM supplemented with 5% FBS (FaDu, SCC-25, Cal27, A253, and PC-3, RV-1) or T-Media (Invitrogen) supplemented with 5% FBS (LNCaP and C4-2) in a humidified 37°C incubator with 5% CO_2_. The mutational status of all the cells used in the screen with the exception of RV-1 prostate cancer cells were verified by OncoMap [29]. RV-1 cells were verified by comparing androgen receptor expression and transcriptional activity to published results. Cells were routinely tested for mycoplasma contamination using MycoAlert (Lonza).

The drugs used for screening were purchased from Calbiochem, lapatinib from L.C. Laboratories, NVP-BEZ235 from Chemitek, AT7867 from Selleck and PD325901 was obtained from Pfizer. All of the primary antibodies used for Western blotting in this study were purchased from Cell Signaling Technologies with the exception of the total and phosphorylated ERK antibodies (Sigma) and the tubulin antibody (EMD Biosciences). The fluorescently labeled secondary antibodies used for Western blotting were from Licor. The antibodies used for flow cytometry were as follows: the anti-rabbit PE-conjugated secondary antibody was from Santa Cruz, the anti-BrdU-FTIC was antibody from BD Biosciences, the anti-PARP-FITC antibody from Fisher Scientific. Bromodeoxyuridine (BrdU) was from Invitrogen.

### Drug combination screening

Cells were grown to 80% confluence, then trypsinized and made into a single cell suspension. BC and HNC cells were resuspended in phenol red-free RPMI 1640 supplemented with 0.5% FBS. CaP cells were resuspended in phenol red-free RPMI 1640 supplemented with 5% charcoal stripped serum (CSS). LNCaP, C4-2, and RV-1 cells were divided into two separate pools. One pool was treated with 0.1 nM R1881. Cells were plated at densities ranging between 3000 and 7000 cells per well in 96 well plates (depending on the rate of growth of the individual cell line) using the BIO-MEK NX workstation. The BIO-MEK NX workstation was used to transfer drug solutions from the master plates with 10X drug concentrations to the cell plates at the final concentration. The cells were incubated for 72 hours at 37°C, 5% CO_2_. After this incubation, the BIO-MEK NX workstation was used to add alamarBlue (Invitrogen) to each plate. The cells were incubated for another 4 hours under the same conditions. Relative cell number was then assessed using a fluorescence plate reader with a 540/25 nm excitation filter and a 620/40 nm emission filter. The data were analyzed by comparing the cytotoxicity caused by combination drug treatment to the predicted additive cytotoxicity calculated using the Bliss independence model [30,31].

### Generation of stable KU-7 p70shRNA cells

The doxycycline-inducible pTRIPZ-RFP-p70shRNA was purchased from Open Biosystems. This vector was transfected into 293-T cells along with the lentiviral packaging and envelope vectors psPAX2 and pMDG by calcium phosphate transfection. Lentivirus was collected two days after transfection and filter sterilized through a 0.24 μm filter. KU-7 cells were infected with lentivirus containing the pTRIPZ-RFP-p70shRNA or mock transduced. Both sets of cells were then exposed to 2.5 μg/mL puromycin until the mock-transduced plate was completely cleared. RFP expression in the KU-7 p70shRNA cells upon 0.2 μg/mL doxycycline treatment was assessed by fluorescence microscopy and flow cytometry

### Flow cytometry

Cells were plated for 24 hours in phenol-red free RPMI-1640 and then treated as described in the text. Six hours prior to collection, cells were pulsed with 15 μg/mL BrdU (when applicable). Both floating and adherent cells were collected and pooled. The cells were fixed with paraformaldehyde and permeabilized with ice-cold methanol and stored in methanol at -20°C until use. Prior to staining, the cells were pelleted in siliconized tubes and washed twice with PBS containing 1% BSA. A partial DNA digestion was then performed with 2M HCl at room temperature for 20 minutes (when applicable). The cells were then blocked using PBS containing 2% donkey serum, 1 % BSA, 0.1% Triton X-100, and 0.05% Tween 20 for 10 minutes. Cells were then stained with the appropriate primary antibody or antibody mixture diluted in PBS with 1% BSA for 1 hour. From this point forward, the cells were kept in the dark in order to preserve the fluorescence signal. Cells were then stained with the appropriate secondary antibody for 30 minutes and then with 1 μg/mL DAPI in PBS. The cells were stored at 4°C overnight and then assayed using a FACSCalibur flow cytometer. Single stained controls were also assayed on the FACSCalibur to serve as compensation controls. Data analysis, including compensation, was performed using Flo-Jo flow cytometry analysis software

### Cell lysis, immunoprecipitation and Western blotting

Cells were plated for 24 hours in phenol-red free RPMI 1640 + 0.5% FBS and then treated as described in the text. Prior to cell lysis, each plate was treated with 1 μM pervanadate and 5 nM Calyculin-A for one minute. The medium was aspirated off and the cells were washed for 30 seconds with ice-cold PBS containing pervanadate and Calyculin A. The PBS was removed and cells were lysed in a Triton-based lysis buffer (1% Triton X-100, 50 mM Tris pH 7.5, 100 mM NaCl, 50mM NaF, and 5 mM EDTA) containing 1 μg/ml pepstatin, 1 μg/ml leupeptin, 1 mM phenylmethylsulfonyl fluoride, 200 μM orthovanadate, 50 mM β-glycerophosphate, and 0.4 μM microcystin. For immunoprecipitation, 1 mg of lysate was treated overnight with total BAD antibody at 4°C. Protein A agarose beads were then added for 4 hours of rocking at 4°C. The beads were collected by centrifugation and washed three times with lysis buffer containing protease and phosphatase inhibitors. The immunoprecipitated protein was then eluted from the beads by boiling in 1X LSB. Western blotting was carried out as previously described [32]. Immunoblots were analyzed using the Odyssey (LICOR Biosciences) imaging system.

### Statistics

The primary method of analysis is 2-way ANOVA for randomized block designs, which were used to control for experiment-to-experiment variation [33]. Contrasts were used to make specific comparisons between groups, and where appropriate, data were transformed to the log scale to facilitate interpretations as fold changes. Synergy was assessed by comparing observed values and values that would be predicted by Bliss-independence [30,31] and by testing for interactions terms in the two-way ANOVA.

## Supplementary Figures


