# Supplementary material for: Combinatorial drug screening identifies compensatory pathway interactions and adaptive resistance mechanisms
Source: Oncotarget. 2013 Apr 10;4(4):622–35. doi: 10.18632/oncotarget.938 (PMC3720609; doi:10.18632/oncotarget.938)
Supplement: Supplementary file 1 [file oncotarget-04-622-s001.pdf]

Combinatorial drug screening identifies compensatory pathway interactions and adaptive resistance mechanisms - Axelrod et al

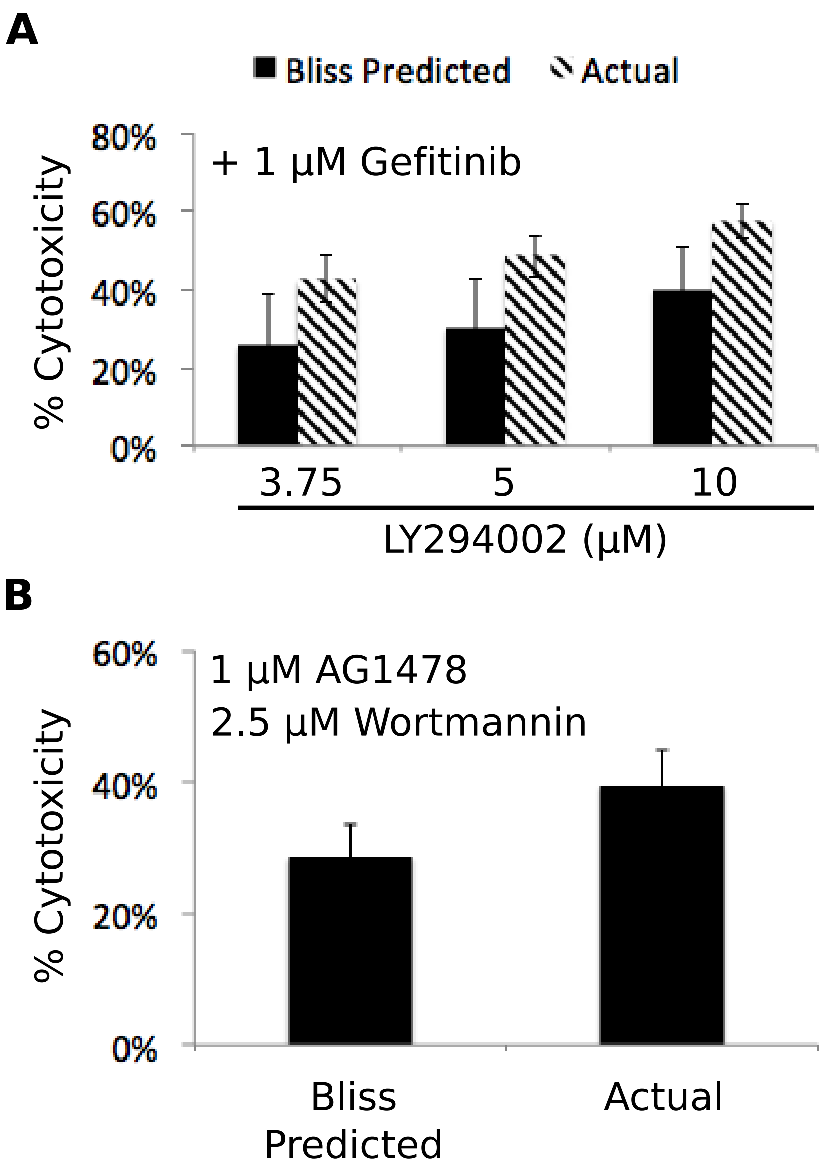

Supplemental Figure S1: Double drug substitutions for lapatinib and BEZ235 maintain the synergy in UMUC-6 cells. A. UMUC-6 cells were treated for 72 hours with the indicated concentrations of gefitinib (substituted for lapatinib) and LY294002 (substituted for BEZ235) or (B) AG1478 (substituted for lapatinib) and wortmannin (substituted for BEZ235). Cell growth was assayed by alamarBlue. Synergistic interaction was determined using the Bliss Additivity model. Bars represent the mean of at least 4 independent experiments and the error bars represent the SEM.

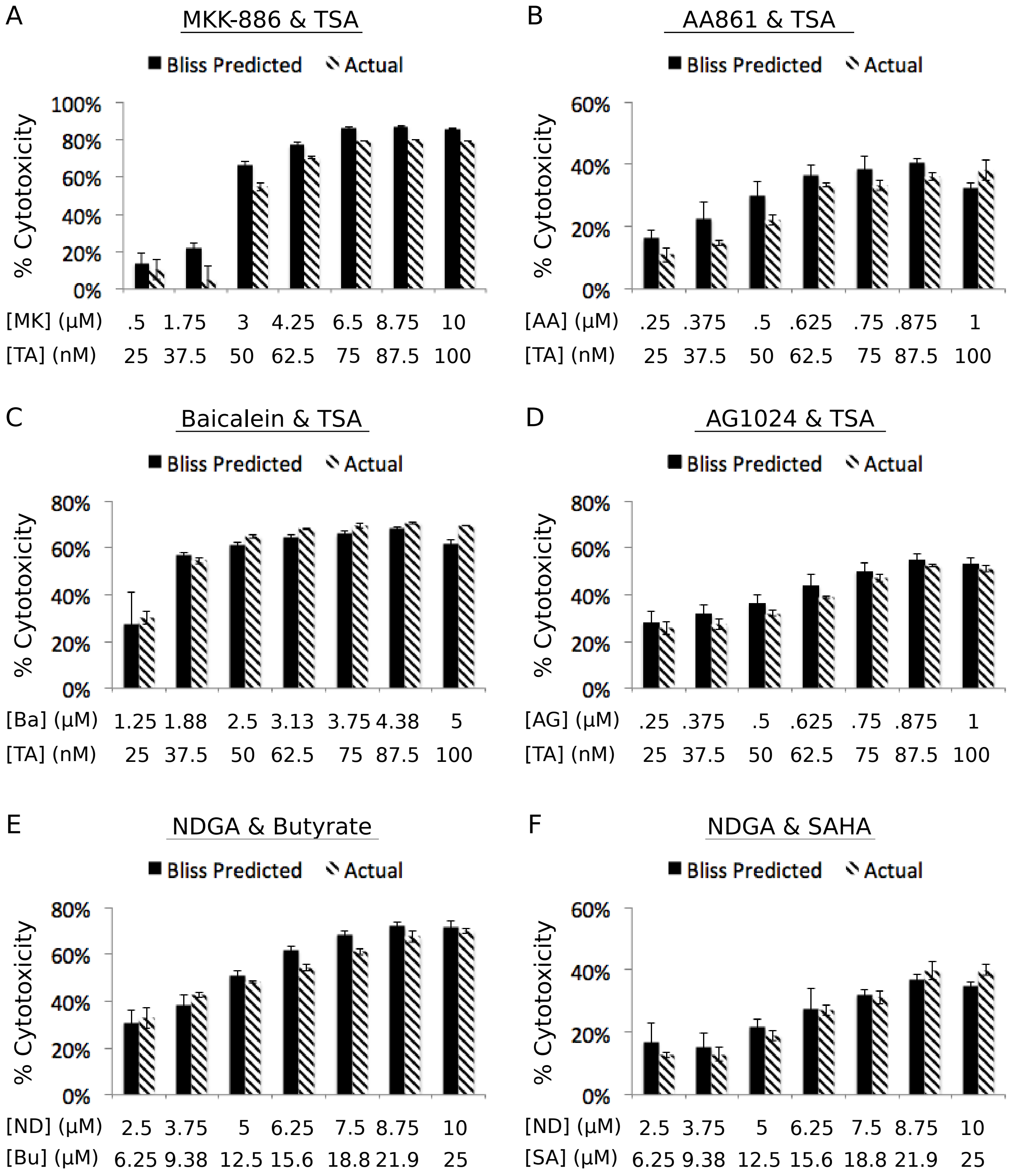

Supplemental Figure S2: Drug substitutions for NDGA and TSA failed to generate synergy in alamarBlue assays in FaDu cells. FaDu (HNC) cells were treated for 72 hours with the indicated drugs and concentrations. (A) MKK-886 (FLAP inhibitor), (B) AA-861 (5-LOX inhibitor), (C) Baicalein (12- and 15-LOX inhibitor) and (D) AG1024 (IGFR inhibitor) were used to substitute for NDGA. (E) Sodium Butyrate and (F) SAHA were used to substitute for TSA. Bars represent the mean and error bars represent the standard deviation of three technical triplicates.

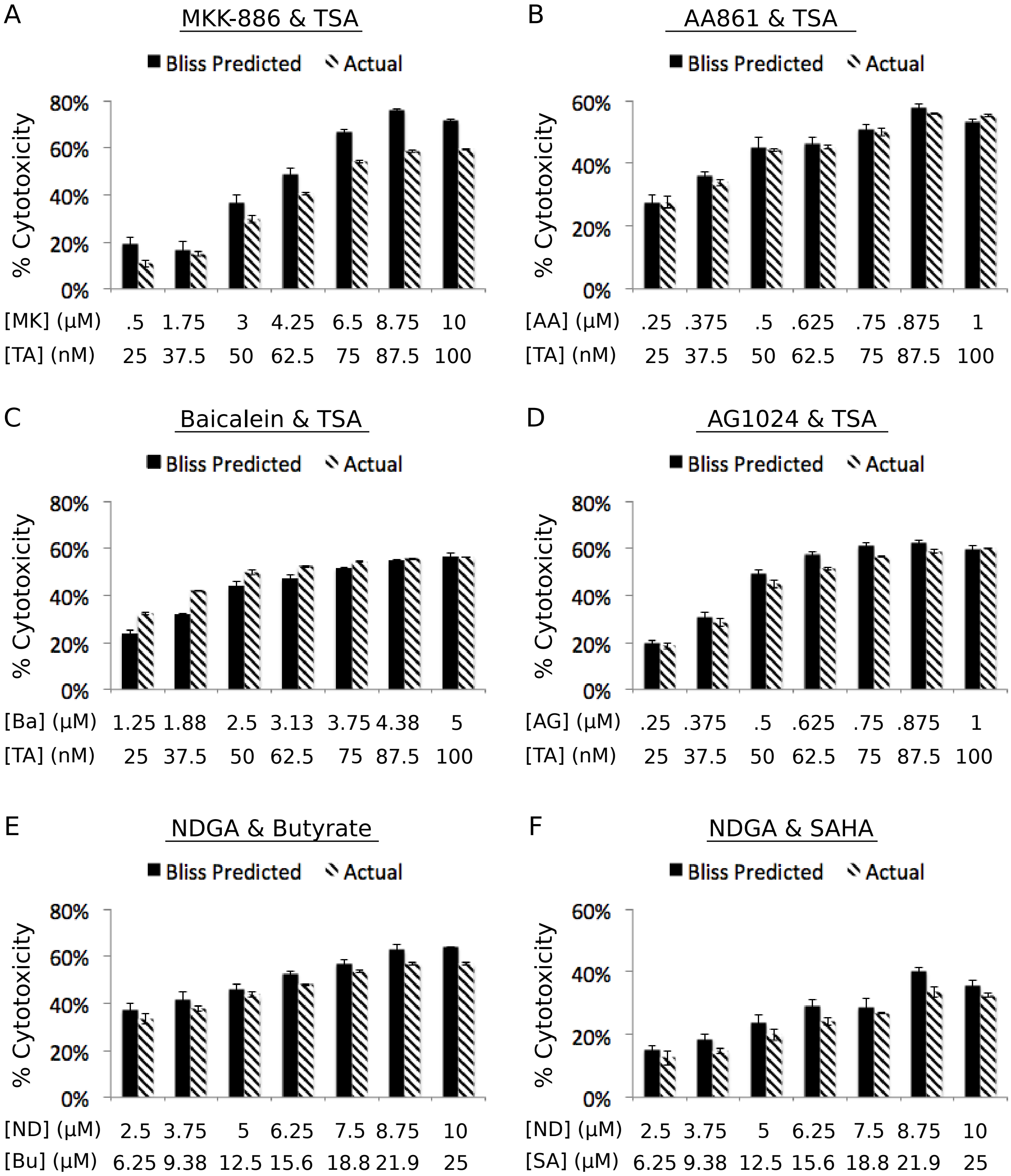

Supplemental Figure S3: Drug substitutions for NDGA and TSA failed to generate synergy in alamarBlue assays in RV-1 cells. RV-1 (CaP) cells were treated for 72 hours with the indicated drugs and concentrations. (A) MKK-886 (FLAP inhibitor), (B) AA-861 (5-LOX inhibitor), (C) Baicalein (12- and 15-LOX inhibitor) and (D) AG1024 (IGFR inhibitor) were used to substitute for NDGA. (E) Sodium Butyrate and (F) SAHA were used to substitute for TSA. Bars represent the mean and error bars represent the standard deviation of three technical triplicates.
